# Influence of maternal anxiety on child anxiety during dental care: cross-sectional study: Influência da ansiedade materna na ansiedade infantil frente ao atendimento odontológico: estudo transversal

**DOI:** 10.1590/1516-3180.2016.027728102016

**Published:** 2017-04-13

**Authors:** Paloma Busato, Raíssa Rigo Garbin, Catielma Nascimento Santos, Luiz Renato Paranhos, Lilian Rigo

**Affiliations:** I Dentistry Student, School of Dentistry, Faculdade Meridional (IMED), Passo Fundo (RS), Brazil.; II Medical Student in the School of Medicine, Universidade de Passo Fundo (UPF), Passo Fundo (RS), Brazil.; III MS. Odontologist, Department of Dentistry, Universidade Federal de Sergipe (UFS), Lagarto (SE), Brazil.; IV PhD. Professor, Department of Dentistry, Universidade Federal de Sergipe (UFS), Lagarto (SE), Brazil.; V PhD. Professor in the School of Dentistry, Faculdade Meridional (IMED), Passo Fundo (RS), Brazil.

**Keywords:** Child behavior, Maternal behavior, Dental anxiety, Dentistry, Manifest anxiety scale

## Abstract

**CONTEXT AND OBJECTIVES:**

Anxiety is usually classified as a disorder of neurotic nature and is often related to contexts of stress, which may include worries, motor tension and autonomic hyperactivity. The aim of this study was to assess the influence of mothers’ anxiety on their children’s anxiety during dental care.

**DESIGN AND SETTING:**

Analytical cross-sectional study conducted at in a private dentistry school in the south of Brazil.

**METHODS:**

Convenience sampling was used. All mothers of children undergoing treatment were invited to participate in this study. Data to investigate anxiety related to dental treatment among the children were collected through applying the Venham Picture Test (VPT) scale. For the mothers, the Corah scale was applied. A self-administered sociodemographic questionnaire with questions about demographic, behavioral, oral health and dental service variables was also used.

**RESULTS:**

40 mother-child pairs were included in the study. The results showed that 40% of the children were anxious and 60% of the mothers were slightly anxious. Local anesthesia was the procedure that caused most anxiety among the mothers, making them somewhat uncomfortable and anxious (60%). Family income higher than R$ 1,577.00 had an influence on maternal anxiety (75.6%). Maternal anxiety had an influence on child anxiety (81.3%).

**CONCLUSION:**

Most of the children showed the presence of anxiety, which ranged from fear of dental care to panic, inferring that maternal anxiety has an influence on children’s anxiety. Dental procedures did not interfere with the mothers’ anxiety, but caused positive feelings, whereas they affected the children more.

## RESUMO

**CONTEXTO E OBJETIVO:**

A ansiedade é geralmente classificada como um transtorno de caráter neurótico, frequentemente relacionado a contextos de estresse variando entre preocupações, tensão motora e hiperatividade autonômica. O objetivo desta pesquisa foi avaliar a influência da ansiedade materna na ansiedade de seu filho durante o atendimento odontológico.

**TIPO DE ESTUDO E LOCAL:**

Estudo analítico transversal realizado em uma faculdade particular do sul do Brasil.

**MÉTODOS:**

Amostragem por conveniência foi utilizada. Todas as mães das crianças em tratamento foram convidadas a participar da pesquisa. A coleta dos dados para verificar a ansiedade relacionada com o tratamento odontológico nas crianças foi realizada a partir da aplicação da escala “Venham Picture Test” (VPT). Para as mães, foi utilizada a escala de Corah. Também se utilizou um questionário sociodemográfico autoaplicativo sobre variáveis demográficas, comportamentais, de saúde bucal e de serviço odontológico.

**RESULTADOS:**

Foram incluídos 40 pares de mães e crianças. Os resultados mostraram que 40% das crianças estavam ansiosas e 60% das mães estavam levemente ansiosas. A anestesia local foi o procedimento que causou mais ansiedade entre as mães, deixando-as um pouco desconfortáveis e ansiosas (60%). Renda familiar maior de R$ 1.577,00 influenciou a ansiedade materna (75.6%). A ansiedade materna influenciou a ansiedade das crianças (81.3%).

**CONCLUSÃO:**

A maioria das crianças apresentou ansiedade, o que variou do medo ao pânico ao atendimento odontológico, inferindo que a ansiedade materna tem influência na ansiedade dos seus filhos. Os procedimentos odontológicos não interferem na ansiedade das mães, atingindo mais as crianças, porém provocam sentimentos positivos.

## INTRODUCTION

Anxiety is usually classified as a disorder of neurotic nature and is often related to contexts of stress, with symptoms that may include worries, motor tension and even autonomic hyperactivity.^1^ Anxiety and fear are common in routine dental practice. Pediatric dentists are better trained to provide smoother child care, which contributes towards a better relationship between patients and professionals.^2^ Historically, the study by Johnson and Baldwin was one of the first to identify a positive and significant correlation between maternal anxiety and the repertoire of behavior of children who were undergoing treatment.^3^ In the 1970s, there were significant improvements regarding equipment, procedures, techniques and materials. However, dental treatment still causes a series of concerns for dentists, especially regarding provision of dental care for children.

Anxiety among patients during their dental treatment is one of the greatest challenges faced by dentists, considering that it hinders implementation of clinical procedures. This situation may lead patients either not to show up or to quit treatment, which usually ends up worsening their oral health condition. Over time, if these patients do not undergo the treatment that they should, the treatment required will become more specialized, with procedures that are more invasive and which also involve higher financial costs.^5^ Difficulties in treating children may lead professionals to feel dispirited and incapable. Hence, it is important to first acknowledge patients’ anxious behavior, so that techniques for achieving clinical security may be applied.^4^

People are not born with anxiety and fear of dental treatment and/or the dentist. This association occurs through the socialization process. Children are as susceptible to anxiety as adults, and their anxiety is derived from peer communication of reported bad experiences or even from threats that parents make.^5^ All of this makes clinical and psychological management more difficult because of children’s different understandings. Therefore, it needs to be possible to work beyond a simple approach of advocating regular visits to dentists, with emphasis on the notion that this is a normal everyday activity and that it may even be enjoyable.

Despite extensive technological advances in dentistry and a search for more humanized services focused on support and comprehensive care, there is still a pattern of thought, especially among the Brazilian population, that associates the dental environment with a place that will cause pain and may generate feelings of fear and anxiety. Such values are transferred from one generation to the next, thus creating a cycle of fear and distress in early childhood. These feelings prevail into adulthood, when they will be reproduced again and transferred to future generations.^5^

Lee et al.^6^ suggested that the anxious behavior of adults during dental treatment may have been acquired through childhood fears, which would therefore require dentists to properly handle child patients. The importance of their study for Brazilian children’s health is that it shows how their anxious behavior during dental treatment and parental influence may be associated, considering that fear of dentists greatly affects children.^7^ This fear also seems to be associated with non-collaborative behavior and lack of visits to a dentist.^6^ Armfield et al.^8^ explored the relationship between dental fear and dental care. They showed that a vicious circle existed, in which people who were very fearful were more prone to delaying their treatment, thereby leading to worsening of their problems and fueling the dental fear that was already present. These occurrences directly involve dental care and negatively affect children’s oral health indicators in Brazil.^9^

The topic of fear and anxiety during dental treatment needs to be researched in several populations, in order to minimize the impact of these factors on oral treatments. Moreover, studies on maternal and child influences, in mother-child pairs, are still scarce in the literature.

## OBJECTIVE

This study aimed to assess the association between maternal anxiety and child anxiety within dental care.

## METHODS

This study was approved by our institution’s Ethics Committee for Research on Human Beings, under protocol 1.096.053. It was conducted in compliance with ethical and legal norms.

### Study design, setting and participants

The present study had a quantitative approach and cross-sectional design. The sample was obtained according to convenience: all mothers of children undergoing treatment at the Children’s Dental Clinic of the Dentistry School of Faculdade Meridional (IMED) were invited to participate in this study, during the months of March and April 2014, as well as their children aged 5 to 10 years. Mothers who were participating in their children’s first visit to a dentist, and children of age groups differing from the study proposal were excluded from the study. All the children were interviewed and were given dental treatment by the same professional, who was a dentistry student. All the children were always accompanied by their mothers, and this formed an inclusion criterion.

### Description of data collection instruments

Data on the children were gathered by applying the modified Venham Picture Test (VPT), which assesses the emotional reactions of children when they choose the drawing of a human figure that best identifies them at that moment. It is considered easy to apply and clear, and little time is required for giving responses.^10^ Originally, the scale presented 42 drawings only with figures of male gender.^10^ Later on, the number of figures was reduced, the female gender was included and the scale went through some changes that made it more practical. These changes included the way in which pairs of figures were presented and the size of heads in relation to the rest of the body.^11^

The questions that the children were asked were standardized and the figures depicted children at the size of half of an A4 sheet of paper (105 mm wide by 98.5 mm long), in color. Drawings of female figures were given to the girls, and male figures to the boys. The test included seven charts with emotional reactions for the different genders.^12^ The figures expressed several reactions, and the children were asked to choose which one most reflected their emotions at that moment, such as: low anxiety (image 1 - neutral), no anxiety (image 2 - happy), and presence of anxiety (image 3 - fearful; image 4 - distressed and crying; image 5 - sad; image 6 - angry; and image 7 - panicky).^12^

Information on the reproducibility of the modified VPT data collection instrument was obtained through applying the questionnaire on a second occasion, to five children who had participated in the pilot study, with a one-week interval between the events. The correlation between the two response times was calculated by means of Spearman’s correlation coefficient. Among the five participants, four (80%) answered the questionnaire identically on the two occasions. The Spearman’s coefficient values varied between 0.07 and 1. Overall, it can be concluded that there was a good correlation between the two times when the mothers answered the questionnaire. Therefore, the reproducibility of this instrument can be considered satisfactory. The mothers and their respective children who participated in the pilot study did not participate in the final study.

The data collection instrument used for the mothers was a self-administered sociodemographic questionnaire, with semi-structured questions that asked for demographic, behavioral, oral health and dental care variables. The questionnaire used was based on another study^5^ with the same variables, which were modified and included in the present study.

In addition, the Corah scale was applied to the mothers, with questions about dental situations, in order to investigate anxiety during dental care.^13^ The Corah scale has four questions about dental situations: “If you had to go to the dentist tomorrow, how would you feel?”; “When you are in the waiting room of the clinic, waiting to be called by the dentist, how do you feel?”; “When you are in the dentist’s chair waiting for him to start the local anesthetic procedures, how you feel?”; and “You are the dentist’s chair, already anesthetized. While waiting for the dentist get the instruments to start the procedure, how does it feel?” For each question, there are five answer options, which are: “quiet, relaxed”; “a little uncomfortable”; “tense”; “anxious and afraid”; and “so anxious or afraid that I start to sweat and feel bad.”

### Description of data collection procedures

The data collection procedures took place in the waiting room before dental care, in a room separated from other patients, in order to maintain the participants’ privacy and ensure the confidentiality of responses.

After the mothers had given their consent, the investigation was directed towards the children in an extroverted manner in which the modified VPT scale was presented as a game, so as to make the children comfortable about participating.

The questions were asked by a single examiner, who used a standardized approach to address the children and apply the instrument; she had previously been trained for this. The examiner was a graduate in educational psychology and had had experience of working with children, which facilitated their understanding of questions.

After the scale had been presented to the children, a standardized question specific for this test was also asked: “All of these children are waiting to enter the dentist’s office. Look at their faces. Which one looks most like you?” If the child presented signs of not understanding the question, it was asked differently, in a manner that was standardized as a second option: “Do you see the faces of these children? Do any of them perhaps look like you at this moment?” If any children refused to participate or claimed not to look like any of the children on the scale, the researcher could motivate the game by choosing one of the faces herself and asking the children to choose theirs.

After the activity with the child had been finished, the sociodemographic questionnaire and the Corah scale were applied to the mother.

### Data analysis

A descriptive data analysis was performed on the results obtained from the modified Venham Picture Test (VPT) that had been applied to the children. Two categories were set up: presence of anxiety or low or no anxiety, according to the code on each figure. The codes ranged from 0 to 6. Code 0 (neutral) represented low anxiety, code 1 (happy) represented no anxiety and codes 2, 3, 4, 5 and 6 represented presence of anxiety. The anxiety-level results obtained from the Corah scale directed towards the mothers were interpreted as follows: patients whose answers totaled less than 5 points were considered to be minimally anxious; from 6 to 10 points, slightly anxious; from 11 to 15 points, moderately anxious; and over 15 points, extremely anxious.

Maternal anxiety was used as the dependent variable, which was categorized as minimally anxious or some degree of anxiety: all mothers with some degree of anxiety were put together in a single group. The independent variables were as follows: child’s age; mother’s age; child’s gender; mother’s education level; family income; mother’s assessment of dental experience; whether the child had already presented some major medical problem; whether the child had already had caries (cavities); whether the child had already had teeth extracted; and the child’s anxiety level.

All data were written down and typed into a specific database for the descriptive and inferential statistical analyses of the present study. Data were electronically processed through the Statistical Package for the Social Sciences software (SPSS), version 17.0. For the present study, it was decided not to perform multivariate analysis but to use bivariate analysis by means of Pearson’s chi-square test (χ^2^). This hypothesis test had the aims of finding dispersion values for each of the nominal variables and assessing associations that might exist among qualitative variables. Thus, the association between the dependent variable (maternal anxiety) and the independent variables was tested at a 5% significance level and with a 95% confidence interval, taking the unilateral hypothesis into consideration. The variables deemed to be associated with the outcome were the ones with significance levels less than or equal to 0.05.

## RESULTS

Over the study period, 40 consecutive children were treated in the service, which resulted in a sample of 40 mother-child pairs.

Table 1 describes the variables relating to the children, among whom the prevailing age group was from 7 to 10 years. Out of the 40 children, 31 (77.5%) had not presented any major medical problems and the majority (70.0%) had already had dental caries. Most of the children (40.0%) showed presence of anxiety (fear and panic) before dental care.

**Table 1.** Distribution of child variables

Variables	n (40)	% (100)
Child’s age (years)		
5-6	10	25.0
7-8	15	37.5
9-10	15	37.5
Child’s gender		
Female	20	50.0
Male	20	50.0
Child had had some major medical problem		
No	31	77.5
Yes	9	22.5
Child had already been to the dentist		
No	0	0
Yes	40	100
Child had already had caries		
No	12	30.0
Yes	28	70.0
Child had already had teeth extracted		
No	23	57.5
Yes	17	42.5
Child’s feeling before dental care		
Neutral	10	25.0
Happy	14	35.0
Fearful	3	7.5
Distressed and crying	2	5.0
Sad	5	12.5
Angry	4	10.0
Panicky	2	5.0
Venham Picture Test result		
Low anxiety	10	25.0
No anxiety	14	35.5
With anxiety	16	40.0

The Corah scale results showed that 16 children (40.0%) were minimally anxious; 20 (50.0%) were slightly anxious; three (7.5%) were moderately anxious; and one (2.5%) was extremely anxious. Table 2 describes the variables relating to the mothers, according to the two categories of mothers’ degrees of anxiety.

**Table 2.** Distribution of maternal variables, according to the two categories of mothers’ degrees of anxiety

Maternal variables	Maternal anxiety			
	Very little		Some level of anxiety	
	n (16)	% (40)	n (24)	% (60)
Mother’s age (years)				
24 to 31	6	46.2	7	53.8
32 to 40	5	31.3	11	68.8
41 to 50	5	45.5	6	54.5
Mother’s education level				
Up to completion of elementary school	5	45.5	6	54.5
High school	9	45.0	11	55.0
Higher education	2	22.2	7	77.8
Monthly family income (in Brazilian reais)				
Less than 788.00	3	18.3	0	0
789.00 - 1,576.00	7	43.8	5	20.8
1,577.00 - 2,364.00	3	18.8	8	33.3
More than 2,364.00	3	18.8	11	45.8
Mother’s assessment of dental experience				
Good	15	46.9	17	53.1
Bad	1	12.5	7	87.5
Mother’s perception regarding child anxiety				
No	7	43.8	9	37.5
Yes	9	56.3	15	62.5
If required to visit the dentist tomorrow				
Ok, I would not mind	16	100.0	14	58.3
I would be slightly concerned	0	0	5	20.8
I would feel great discomfort	0	0	2	8.3
I would be afraid of what may happen	0	0	2	8.3
I would be very apprehensive and would not sleep well	0	0	2	4.2
Waiting to be called by the dentist in the waiting room				
Calm, relaxed	14	87.5	9	37.5
A little uncomfortable	2	12.5	6	25.0
Stressed	0	0	4	16.7
Anxious or afraid	0	0	5	20.8
Very anxious and afraid, starting to sweat and feeling bad	0	0	0	0
During local anesthesia				
Calm, relaxed	14	87.5	2	8.3
A little uncomfortable	2	12.5	7	29.2
Stressed	0	0	9	37.5
Anxious or afraid	0	0	5	20.8
Very anxious and afraid, starting to sweat and feeling bad	0	0	1	4.2
After anesthesia, waiting for the dentist to pick up instruments				
Calm, relaxed	16	100.0	12	50.0
A little uncomfortable	0	0	8	33.3
Stressed	0	0	3	12.5
Anxious or afraid	0	0	1	4.2
Very anxious and afraid, starting to sweat and feeling bad	0	0	0	0

### Inferential data analysis

From the statistical analysis, it could be seen that neither the sociodemographic characteristics (variables of child’s age and gender) nor the mother’s age and education level were associated with the dependent variable (maternal anxiety). The variables of mother’s dental experience and child’s experience (of major medical problems, dental caries or tooth extraction) were also not related to the dependent variable.

However, there were significant associations between maternal anxiety and the variables of family income (P = 0.030) and child’s anxiety (P = 0.026). These results showed that family income higher that R$ 1,577.00 had an influence on maternal anxiety (75.6%). Moreover, there was a relationship between mothers who presented some level of anxiety and children with anxiety (81.3%), with P = 0.026. These results are shown in Table 3.

**Table 3.** Bivariate analysis on sociodemographic variables associated with maternal anxiety and its relationship to child anxiety

Independent variables	Maternal anxiety						P
	Minimally anxious		Some level of anxiety		Total		
	n	%	n	%	n	%	
Child’s age (years)							
5 - 6	5	50.0	5	50.0	10	100.0	0.707
7 - 8	5	33.3	10	66.7	15	100.0	
9 - 10	6	40.0	9	60.0	15	100.0	
Total	16	40.0	24	60.0	40	100.0	
Mother’s age (years)							
24 to 31	6	46.2	7	53.8	13	100.0	0.65
32 to 40	5	31.3	11	68.8	16	100.0	
41 to 50	5	45.5	6	54.5	11	100.0	
Total	16	40.0	24	60.0	40	100.0	
Child’s gender							
Female	9	45.0	11	55.0	20	100.0	0.374
Male	7	35.0	13	65.0	20	100.0	
Total	16	40.0	24	60.0	40	100.0	
Mother’s education level							
Up to completion of elementary school	5	45.5	6	54.5	11	100.0	0.465
High school	9	45.0	11	55.0	20	100.0	
Higher education	2	22.2	7	77.8	9	100.0	
Total	16	40.0	24	60.0	40	100.0	
Monthly family income (in Brazilian reais)							
788,00-1.576,00	10	79.15	5	41.7	15	100.0	0.030
1,577.00 or more	6	24.35	19	75.6	25	100.0	
Total	16	40.0	24	60.0	40	100.0	
Mother’s assessment of dental experience							
Good	15	46.9	17	53.1	32	100.0	0.082
Bad	1	12.5	7	87.5	8	100.0	
Total	16	40.0	24	60.0	40	100.0	
Whether the child had already presented major medical problems							
No	14	45.2	17	54.8	31	100.0	0.200
Yes	2	22.2	7	77.8	9	100.0	
Total	16	40.0	24	60.0	40	100.0	
Whether the child had already had caries							
No	3	25.0	9	75.0	12	100.0	0.181
Yes	13	46.4	15	53.6	28	100.0	
Total	16	40.0	24	60.0	40	100.0	
Whether the child had already had teeth extracted							
No	9	39.1	14	60.9	23	100.0	0.576
Yes	7	41.2	10	58.8	17	100.0	
Total	16	40.0	24	60.0	40	100.0	
Child’s anxiety							
Little or none	13	54.2	11	45.8	24	100.0	0.026
Some anxiety	3	18.8	13	81.3	16	100.0	
Total	16	40.0	24	60.0	40	100.0	

## DISCUSSION

The modified VPT showed that most of the children (40%) were anxious or a little anxious (25%) before dental treatment. Anxiety is frequently correlated with dental treatment because the pain and emotional reactions to this treatment are seen by many patients as threatening their wellbeing.^2^ One study that assessed dental fear levels, states of anxiety and physiological distress among children older than six years of age and their mothers during pediatric dental procedures concluded that maternal anxiety before children’s dental treatment was significantly associated with children’s fear of dental treatment.^14^

Age is one of the factors with a higher impact on the presence of anxiety among children. Younger children tend to be more afraid of the unknown and of abandonment. However, the present study showed that children aged 7 to 10 years were the ones who most presented some level of anxiety. This may be linked to the possibility that because they were older, they may have had previous painful or distressful experiences relating to dental care. In addition, children of this age have a higher level of attention and cognition, and may have correlated the negative experiences of members of their families with their own experiences.

Maternal anxiety was measured in four categories, among which most mothers were classified as slightly anxious (50%) or minimally anxious (40%). These data are corroborated by the results from other studies,^5^ in which the majority of legal guardians of children showed low or slight anxiety during dental care. This characterizes the new profile of dental patients, who have become detached from rooted concepts about dental treatment panic and have start to regard it just as a regular procedure required for oral health maintenance.

From the Corah scale, the present study showed that what makes mothers feel stressed (22.5%) and uncomfortable (22.5%) is the procedure of injected anesthesia. In other study, states of anxiety and physiological distress levels were significantly higher among mothers before their children’s dental treatment but not afterwards.^14^ Soares et al.^15^ showed that 54.8% of the children whose family income was higher than two minimum wages did not present anxiety in relation to dental care. The present study showed a statistically significant association between maternal anxiety and family income. Among the mothers with family income higher than R$ 1,577.00, the majority (75.6%) presented some level of anxiety. This may be explained by the greater ease of access to information on dental procedures among mothers with higher family income.^5^

One of the most controversial points is mothers’ presence in the dental office with their children during dental care.^16^ Some authors have stated that child anxiety is associated with maternal anxiety, and that this relationship may result in non-collaborative attitudes among children.^4^^,^^17^ The present study showed that there was a significant association between maternal anxiety and child anxiety, in which the majority of anxious children (81.3%) had mothers with some level of anxiety. This corroborates the findings from another study that aimed to investigate the effects of sociodemographic factors and maternal anxiety levels on the behavior of children undergoing surgery, which concluded that maternal knowledge and experience of anesthesia, and high levels of maternal anxiety, may be related to increased anxiety among children undergoing surgery.^4^ Lack of psychological preparation for children undergoing dental care tends to cause failures of treatment efficiency and makes success impossible.^18^

The present study had some limitations. Firstly, we only investigated children aged 5 to 12 years. Younger children might have other feelings about pictures that are presented to them. In addition, a larger sample of both mothers and children would have allowed inferential statistical analysis with greater extrapolations of the information and conclusions. Future studies should take these limitations into consideration in reaching new conclusions.

## CONCLUSION

Based on the results from this study and taking into account that the conclusions from this study are limited by the small number of mother-child pairs, it is possible to conclude that:

1. Most of the children showed some anxiety, ranging from feelings of fear to feelings of panic regarding dental care;
2. Most of the mothers were slightly anxious and the dental procedure that caused most anxiety was the expectation of local anesthesia;
3. Mothers with higher family income were more anxious;
4. Maternal anxiety had an influence on child anxiety.

This information is important in relation to adequate training for dental care professionals, especially for those involved in dental care for children. The psychological aspects and subjective issues of such situations need to be thought of as being as essential as the technical focus.
